# Drug resistant bacteria in perianal abscesses are frequent and relevant

**DOI:** 10.1038/s41598-022-19123-6

**Published:** 2022-09-01

**Authors:** Fabienne Bender, Lukas Eckerth, Moritz Fritzenwanker, Juliane Liese, Ingolf Askevold, Can Imirzalioglu, Winfried Padberg, Andreas Hecker, Martin Reichert

**Affiliations:** 1 Department of General, Visceral, Thoracic, Transplant and Pediatric Surgery, University Hospital of Giessen, Rudolf-Buchheim Strasse 7, 35390 Giessen, Germany; 2 Institute of Medical Microbiology, Justus-Liebig-University of Giessen, Schubertstrasse 81, 35392 Giessen, Germany; 3 German Center for Infection Research (DZIF), Site Giessen-Marburg-Langen, Justus-Liebig-University of Giessen, Schubertstrasse 81, 35392 Giessen, Germany

**Keywords:** Gastrointestinal diseases, Dysbiosis, Bacterial infection, Outcomes research

## Abstract

Perianal abscesses are frequent diseases in general surgery. Principles of standard patient care are surgical drainage with exploration and concomitant treatment of fistula. Antiinfective therapy is frequently applied in cases of severe local disease and perianal sepsis. However, the role of microbiologic testing of purulence from perianal abscesses is disputed and the knowledge concerning bacteriology and bacterial resistances is very limited. A retrospective cohort study was performed of consecutive patients (≥ 12 years of age) from a tertiary care hospital, who underwent surgical treatment for perianal abscess from 01/2008 to 12/2019. Subdividing the cohort into three groups regarding microbiological testing results: no microbiological testing of purulence (No_Swab, n = 456), no detection of drug resistant bacteria [DR(−), n = 141] or detection of bacteria with acquired drug resistances from purulence [DR(+), n = 220]. Group comparisons were performed using Kruskall–Wallis test and, if applicable, followed by Dunn´s multiple comparisons test for continuous variables or Fishers exact or Pearson’s X^2^ test for categorical data. Fistula persistence was estimated by Kaplan Meier and compared between the groups using Log rank test. Corralation analysis between perioperative outcome parameters and bacteriology was performed using Spearman´s rho rank correlation. Higher pretherapeutic C-reactive protein (p < 0.0001) and white blood cell count (p < 0.0001), higher rates of supralevatoric or pararectal abscesses (p = 0.0062) and of complicated fistula-in-ano requiring drainage procedure during index surgery (p < 0.0001) reflect more severe diseases in DR(+) patients. The necessity of antibiotic therapy (p < 0.0001), change of antibiotic regimen upon microbiologic testing results (p = 0.0001) and the rate of re-debridements during short-term follow-up (p = 0.0001) were the highest, the duration until definitive fistula repair was the longest in DR(+) patients (p = 0.0061). *Escherichia coli*, *Bacteroides*, *Streptococcus* and *Staphylococcus* species with acquired drug resistances were detected frequently. High rates of resistances against everyday antibiotics, including perioperative antibiotic prophylaxis were alarming. In conclusion, the knowledge about individual bacteriology is relevant in cases of complex and severe local disease, including locally advanced infection with extended soft tissue affection and perianal sepsis, signs of systemic inflammatory response as well as the need of re-do surgery for local debridements during short-term and fistula repair during long-term follow-up. Higher rates of acquired antibiotic resistances are to be expected in patients with more severe diseases.

## Introduction

Perianal abscesses are one of the most frequent diseases in general emergency surgery. Perianal abscesses are frequently of cryptoglandular origin and are commonly complicated by the presence of fistula-in-ano depending on the localization^1–4^. Surgical drainage procedures with exploration of fistula are treatment principles in the acute care setting, but patients with a more severe infectious disease affecting the perianal and perineal region as well as signs of systemic inflammation are frequently in need of an additional antiinfectious therapy^1–3,5,6^. However, the efficacy including the impact on postoperative (antibiotic) management and outcome in general of microbiological testing from purulence of perianal abscesses obtained during drainage surgery is disputed in current literature^7–9^. Vice versa, microbiological testing is still recommended in patients with clinical concerns regarding unfavourable outcomes and a more severe disease^10^. Nevertheless, no clear evidence is provided in literature for microorganisms underlying the abscess. But, some authors report specific bacterial culture for patients having some chronic diseases including diabetes mellitus and immuno-suppression or suffering from fistula-in-ano underlying the abscess ^7,8,11–13^. The same holds true for the detection of acquired drug resistances in the bacteria investigated by culture^12,14^. Both the frequency of bacterial strains as well as the most common antimicrobial drug resistances to be expected in microbiological examination from perianal abscess pus might be of major importance in perioperative care especially of patients with a more complicated and severe disease requiring a multimodal treatment approach. The aim of this retrospective study is to gain evidence for microbiological testing of perianal abscess pus, to evaluate pathogens and respective drug resistances to be expected in purulence of perianal abscesses as well as to define patients, in whom microbiological testing of perianal abscess pus is useful and effective with regard to postoperative management.

## Methods

From 01/2008 to 12/2019 all consecutive patients (≥ 12 years of age), who underwent surgical treatment at the University Hospital of Giessen for perianal abscess (i.e. surgical abscess drainage or local abscess excision both with exploration for an accompanying perianal fistula-in-ano and, if present, primary excision or drainage of the fistula) as well as for extended surgical tissue excision for advanced perianal/perineal soft tissue infection originated from perianal abscesses were retrospectively evaluated and included in this study.

Patient data were analyzed retrospectively from the prospectively maintained institutional database. The present work focused mainly on bacteriology of intraoperatively obtained swabs from purulence of perianal abscesses and on (acquired) drug resistances of the detected microorganisms. Intermediate effectiveness of the antibiotic was not judged as drug resistance. Two microbiologists independently reviewed bacterial culture results and susceptibility testing for intrinsic or aquired drug resistances regarding the current EUCAST (The European Committee on Antimicrobial Susceptibility Testing) breakpoint tables for interpretation of minimum inhibitory concentrations and zone diameters, v11.0, 2021, as well as intrinsic resistance and unusual phenotypes, v3.2, 2020, http://www.eucast.org. Isolates with at least one detected acquired resistance were interpreted as “drug resistant", whereas intrinsic resistances of the detected bacteria, indicated by the respective EUCAST documents, were filtered out. Bacteria with only intrinsic resistances were not interpreted as being "drug resistant".

Analysis of resistances against cefuroxime and metronidazole—both being important drugs for routine perioperative antibiotic prophylaxis in colo-rectal surgery—was performed independently from acquired or intrinsic origin. Detected isolates were furthermore classified according to the ESKAPE definition, which includes the highly virulent and frequently drug resistant pathogens: *Enterococcus faecium*, *Staphylococcus aureus*, *Klebsiella pneumoniae*, *Acinetobacter baumanii*, *Pseudomonas aeruginosa*, *Enterobacter* sp.^15^.

The duration of postoperative hospitalization and duration from index surgery until surgical repair of the fistula were interpreted as surrogate parameters for postoperative outcome. Furthermore, general patient characteristics, preoperative systemic markers of infection including C-reactive protein (CRP) values and white blood cell (WBC) counts in peripheral blood, surgical procedure characteristics, postoperative patient outcome, including re-do surgery, recurrence and the application of postoperative antibiotics were evaluated.

### Surgery and perioperative patient care

Perianal abscesses are treated as surgical emergencies. Therapeutic strategies follow the German guidelines for anal abscess and cryptoglandular fistula^16^. Surgical drainage procedure or excision of the abscess are treatment standards, followed by fistula exploration with care during index surgery. Primary fistulectomy is routinely performed during index surgery in cases of superficial fistulas by experienced surgeons. In cases of unclear findings or transsphincteric and higher located fistulas, an initial loose seton placement for drainage of the fistula during index surgery is performed followed by definitive fistula repair in a second surgical procedure after 4–6 weeks or at the earliest convenience beyond the infectious situation by trained surgeons.

During abscess drainage surgery, swabs from the purulence were obtained upon own judgement of the surgeon. Hence, swabs from abscess pus and infected tissue are regularly harvested in cases of more complicated and severe perianal disease, including a locally advanced infection with extended soft tissue affection, whereas, in patients with a mild and uncomplicated disease swabs are not obtained in clinical routine.

All patients conducted to surgical perianal abscess drainage procedure receive perioperative single-shot antibiotic prophylaxis with cefuroxime and metronidazole intravenously. However, antibiotic therapy is not routinely continued postoperatively. Indications for postoperative antibiotic therapy after surgical drainage of perianal abscesses with or without detection and consecutive drainage or excision of fistulas are complicated perianal infection and perianal as well as perineal sepsis with locally advanced phlegmonous or gangrenous soft tissue infection. Otherwise, patients rinse the perianal wounds by themselves and are routinely discharged as soon as possible on postoperative day one or two.

### Statistical analyses

The patient cohort was subdivided into three groups regarding microbiological swab examination and diagnosis of drug resistant (DR) bacteria: (1) patients in whom microbiological swab was not obtained from the abscess intraoperatively (No_Swab group: n = 456), (2) patients with intraoperative microbiological swab without detection of DR bacteria [DR(−) group: n = 141] or (3) patients with intraoperative swab and detection of acquired drug resistances in bacteria during microbiological examination [DR(+) group, n = 220].

Statistical analyses were performed using GraphPad Prism (Version 9 for Windows, GraphPad Software, San Diego, CA, USA, www.graphpad.com). Retrospective availability of presented data was > 96%. For descriptive statistics, categorical data were analyzed using Fishers exact or Pearson’s X^2^ test. Group comparisons of continuous variables were performed by Kruskall–Wallis test for global effects between the three groups and, if applicable, by Dunn’s multiple comparisons test of each group.

For patients with perianal fistula found during index surgery with abscess drainage, the persistence of fistula, i.e. the duration from index surgery to definitive fistula repair surgery, was calculated by Kaplan Meier estimation. Patients with an initial drainage of the fistula during index surgery, but, who were lost in follow-up were censored from this analysis upon the last contact. This is indicated in Kaplan–Meier curves by vertical ticks. The day of fistula surgery indicated the “event”. If the fistula was approached during index surgery through any kind of fistulectomy, postoperative day “0” was anticipated as the “event”. Log rank test was used for Kaplan–Meier curve comparisons between the groups of patients.

Spearman’s rho rank correlation was used to determine statistical dependences in either the whole patient cohort or in patients with microbiological abscess swab examination between patient characteristics, microbiological culture results and postoperative outcome indicators. Results are given as the Spearman’s rank correlation coefficient (r_SP_) and respective significances.

Heatmaps display correlation coefficients between the respective variables in either the whole patient cohort or exclusively the patients with abscess swab examinations as well as the ratio of acquired resistances of the microorganisms detected in the study.

Data are given in mean ± standard deviation for continuous variables as well as n (%) for categorical data; p-values ≤ 0.05 indicate statistical significance. Because of the exploratory character of the study no adjustments of p-values were performed.

### Ethics approval

This exploratory, retrospective single-center cohort study was performed in accordance with the latest version of the Declaration of Helsinki and was approved by the local ethics committee of the medical Faculty of the University of Giessen (approval No.66/19). Due to the retrospective nature of the study, need for written informed consent was waived by the ethics committee. The data are collected, the manuscript is written and submitted in accordance with the COPE and STROBE guidelines. All patients were treated according with the institutional standard-of-care.

## Results

### Patient cohort and characteristics

817 patients underwent surgical drainage procedure for perianal abscess and were included into the data analysis [No_Swab: n = 456, DR(−): n = 141 and DR(+): n = 220]. Male gender was overrepresented in all groups. More patients from the DR(+) group had evidences for any kind of immunosuppression and suffered from diabetes (Table 1).

**Table 1:** Patient characteristics and perioperative outcome.

Variable	No_Swab-group n = 456	Swab-groups		p-value
		DR(−) n = 141	DR(+) n = 220	
Male gender	324 (71.1%)	107 (75.9%)	176 (80.0%)	0.0398
Age (years)	44.0 ± 14.4^a^	40.6 ± 14.6^a^	43.4 ± 16.8	0.0346
Body mass index (kg/m^2^)	28.2 ± 6.6	27.4 ± 7.1	27.7 ± 7.5	0.0525
Diabetes mellitus	31 (6.8%)	6 (4.3%)	26 (11.8%)	0.0173
Active smoking	148 (32.5%)	64 (45.4%)	69 (31.4%)	0.0100
Chronic pulmonal disease	30 (6.6%)	8 (5.7%)	24 (10.9%)	0.0881
Coronary artery disease	16 (14.4%)	3 (2.1%)	11 (5.0%)	0.3531
Arterial hypertension	95 (20.8%)	24 (17.0%)	51 (23.2%)	0.3717
CIBD	48 (10.5%)	9 (6.4%)	21 (9.5%)	0.3428
Systemic Immunsuppression^b^	15 (3.3%)	6 (4.3%)	21 (9.5%)	0.0023
Surgery ≤ 12 h after initial presentation	260 (57.0%)	102 (72.3%)	151 (68.6%)	0.0005
Supralevatoric or pararectal abscess	9 (2.0%)	5 (3.5%)	15 (6.8%)	0.0062
Gangrenous tissue infection^c^	0	1 (0.7%)	13 (5.9%)	< 0.0001
Detection of fistula during index surgery	316 (69.3%)	70 (49.6%)	120 (54.5%)	< 0.0001
Primary fistula drainage	215 (68.0%)	58 (82.9%)	105 (87.5%)	< 0.0001
Primary fistulectomy	101 (32.0%)	12 (17.1%)	15 (12.5%)	
Stool deviation/Stoma rate	3 (0.7%)	1 (0.7%)	13 (5.9%)	< 0.0001
Postoperative antibiotic therapy	71 (15.6%)	46 (32.6%)	87 (39.5%)	< 0.0001
Change of antibiotic therapies	0	3 (2.1%)	9 (4.1%)	0.0001
Postoperative intensive care	4 (0.9%)	0	11 (5.0%)	0.0002
Re-do surgery (short-term follow-up)	13 (2.9%)	9 (6.4%)	24 (10.9%)	0.0001
Overall recurrency^d^	91 (20.0%)	26 (18.4%)	40 (18.2%)	0.8323
30 day mortality	0	0	2 (0.9%)	0.0659

^a^Indicates significant differences between the groups in Dunn’s multiple comparison. ^b^Including chemotherapy within 8 weeks before abscess surgery. ^c^At index surgery or re-do surgery. ^d^Overall recurrence includes patients with recurrent perianal abscess in long-term follow-up after index surgery or perianal abscess in the patient’s history. *CIBD* Chronic inflammatory bowel disease.

**Figure 1:**
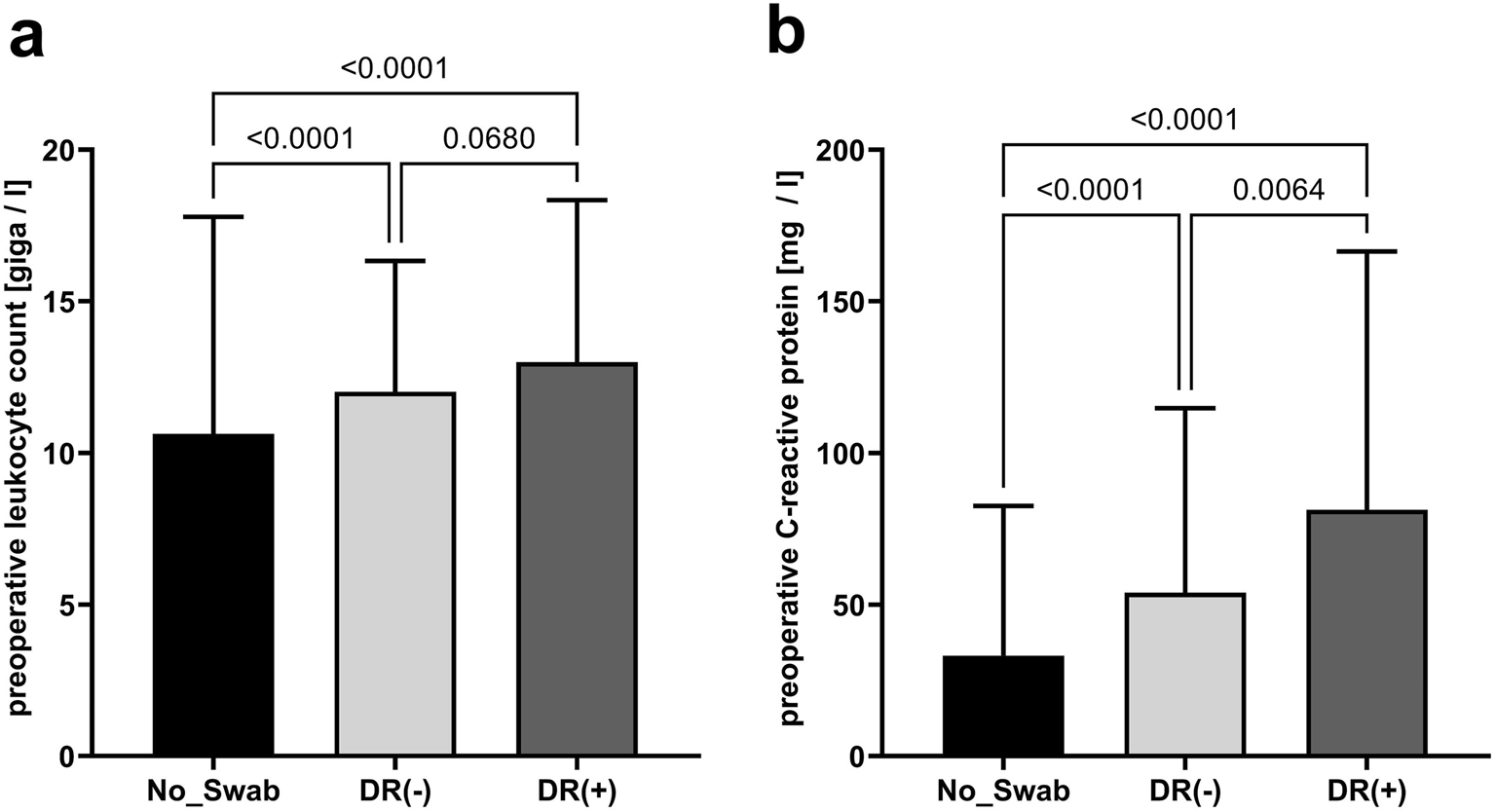
Preoperative markers for systemic inflammation. (**a**) preoperative white blood cell count in peripheral blood (p_[Kruskall–Wallis]_ < 0.0001) and (**b**) preoperative C-reactive protein (p_[Kruskall–Wallis]_ < 0.0001).

**Figure 2:**
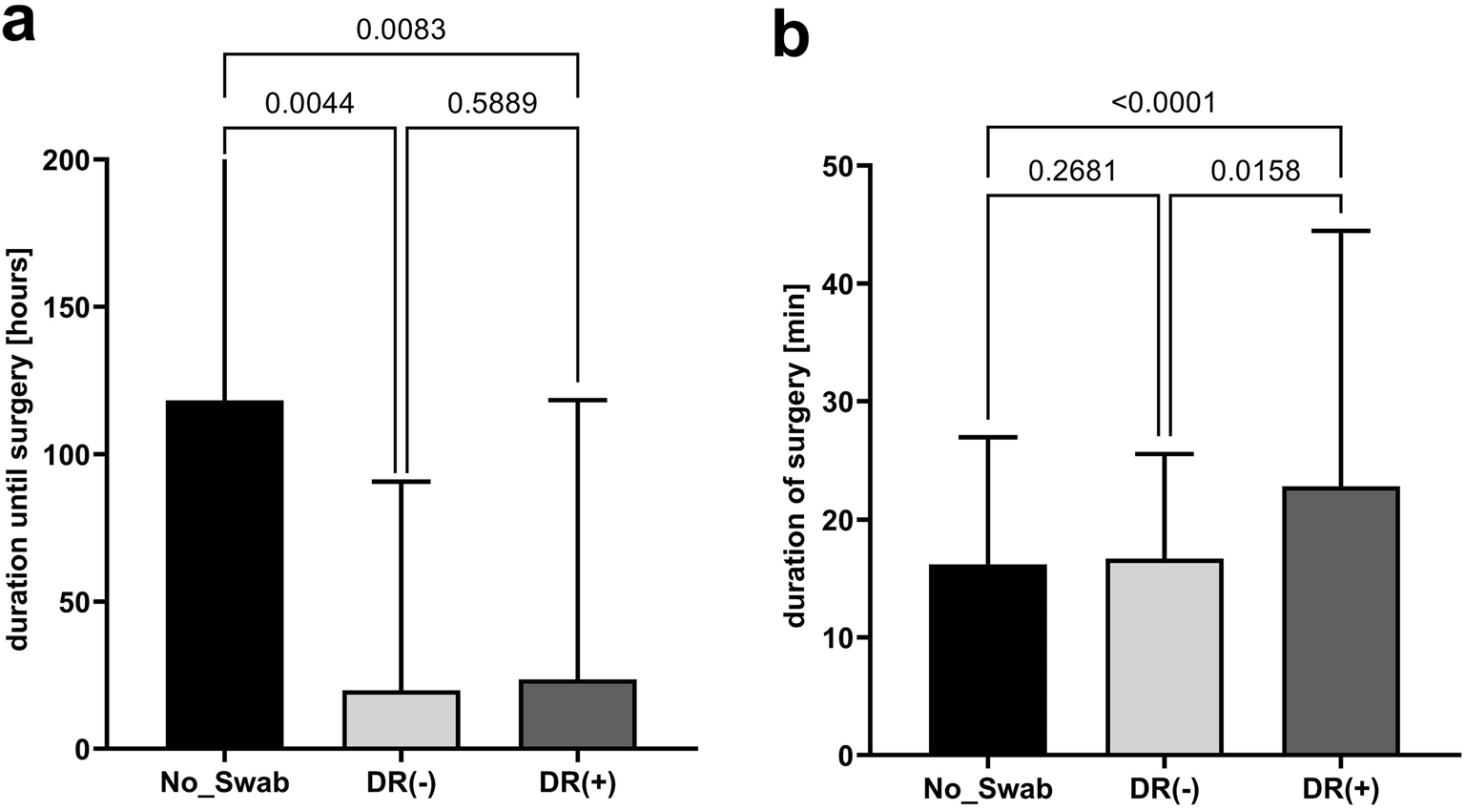
Perioperative outcome. (**a**) Duration from presentation until surgery (p_[Kruskall–Wallis]_ = 0.0027) and (**b**) duration of abscess drainage surgery (p_[Kruskall–Wallis]_ < 0.0001).

In 26 patients from the DR(−) group neither bacteria nor fungi were detected in microbiologic analyses of swabs from the abscesses. However, polybacterial culture (> 1 pathogen) was detected more frequently in abscess swabs from patients of the DR(+) group, containing ESKAPE pathogens in a considerably high percentage (Table 2). Reflecting a more complex and severe infection, serum markers for systemic inflammation and infection, including CRP and WBC in peripheral blood, were the highest in DR(+) patients (Fig. 1). In this line, patients from the DR(+) group suffered clinically more frequently from a locally more advanced disease (i.e. supralevatoric or pararectal abscess formation) and the infection was classified more commonly as being gangrenous during either index or re-do surgery in short-term follow-up, which—vice versa—is a condition urging locally extended tissue resection (Table 1). Furthermore, a significantly longer duration of the index surgical procedure as well as shorter preoperative in-hospital stay might be interpreted as signs for clinical disease complexity or severity and consecutively also reflect the extend of surgery and the emergency character of surgical drainage procedure, respectively, in the DR(+) group especially in comparison with patients from the No_Swab group (Table 1 and Fig. 2).

**Table 2:** Bacteriology in patients without DR(−) or with detection of acquired drug resistances DR(+).

Variable	Swab-groups		p-value
	DR(−) n = 141	DR(+) n = 220	
**Number of detected germs**			< 0.0001
0	26 (18.4%)	–	
1	84 (59.6%) ^*^	99 (45.0%)	
2	25 (17.7%)	82 (37.3%)***	
3	4 (2.8%)	37 (16.8%)*	
4	2 (1.4%)	2 (0.9%)*	
n patients with ESKAPE^a^ bacteria	87 (61.7%)	157 (71.4%)	0.0652
n patients with > 1 ESKAPE bacteria	4 (2.8%)	35 (15.9%)	< 0.0001
**Number of bacteria with acquired drug resistancies (n bacteria)**	–		
1		173	
2		40	
3		7	
4		0	
Patients with bacteria with resistances against cefuroxime and metronidazole^b^	12 (8.5%)	44 (20%)	0.0029
**Gram+**			
*Streptococcus* spp.	16 (11.3%)	59 (26.4%)	0.0003
*Staphylococcus* spp.	5 (3.5%)	27 (12.7%)	0.0041
*Enterococcus* spp.	7 (5.0%)	10 (4.5%)	1
**Gram−**			
*Escherichia coli*	73 (51.8%)	130 (59.1%)	0.1923
*Klebsiella* spp.	4 (2.8%)	15 (6.8%)	0.1457
*Proteus* spp.	0	12 (5.5%)	0.0043
*Citrobacter* spp.	2 (1.4%)	3 (1.4%)	1
*Pseudomonas aeroginosa*	2 (1.4%)	2 (0.9%)	0.5635
**Anaerob**			
*Bacteroides* spp.	12 (8.5%)	54 (24.5%)	< 0.0001
*Prevotella* spp.	3 (2.1%)	7 (3.2%)	0.7461

^* ^Asterisks indicating the number of cases with detection of funghi from intraoperatively obtained abscess swabs. ^a ^Although not intended to classify community-acquired infections, the ESKAPE (*Enterococcus faecium*, *Staphylococcus aureus*, *Klebsiella pneumoniae*, *Acinetobacter baumanii*, *Pseudomonas aeruginosa*, *Enterobacter* sp.) definition was used. ^b^Either acquired or intrintrinsic resistances against cefuroxime and metronidazole.

### Perioperative outcome

**Figure 3:**
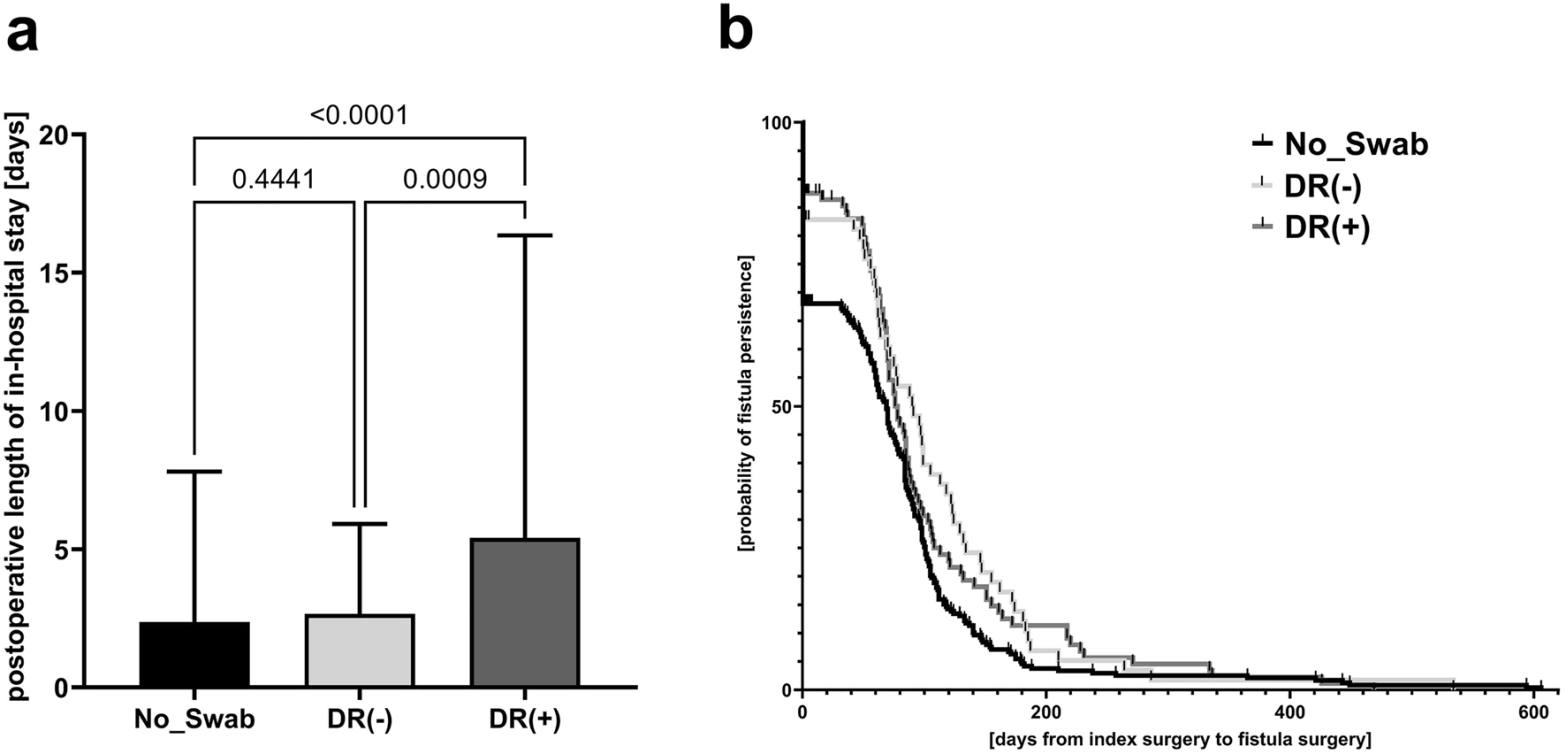
Postoperative outcome. (**a**) duration of postoperative in-hospital stay (p_[Kruskall–Wallis]_ < 0.0001) and (**b**) calculation of the fistula-in-ano persistance (i.e. duration from index surgery to definitive fistula repair surgery) by Kaplan–Meier estimation. Censored data are indicated by vertical ticks. Differences between the groups are indicated by p = 0.0061 in Log rank test.

The frequency of re-do surgery for repeated local debridement as well as the rate for deviating stoma to locally protect extended perianal and perineal wounds in perineal sepsis cases were markedly increased in the DR(+) group. 32.6% and 39.5% of patients from DR(−) and DR(+), respectively, versus 15.6% of No_Swab patients (p < 0.0001) needed postsurgical antibiotic therapy. Notably, 4.1% of DR(+) patients needed changes of the antibiotic regimen during postoperative follow-up versus 2.1% and none of the patients from the DR(−) and No_Swab group (p = 0.0002), respectively (Table 1). The length of postoperative hospitalization after index surgery as a surrogate outcome parameter was the longest in DR(+) patients (Fig. 3a). Although simple fistula, which were primarily excised during index surgery with abscess drainage, were found more frequently in patients from the No_Swab group, vice versa most fistula from patients of the DR(+) group required primary drainage procedure during index emergency surgery. In the long term follow-up, the duration until surgery to the definitive repair of the fistula and consecutively the duration of fistula-in-ano persistence was significantly longer in patients with positive bacteriology compared to the respective patients from the No_Swab group (p = 0.0061). As depicted by Kaplan–Meier curves, this effect was most obviously beyond day 180 after index surgery in DR(+) patients with initially drained perianal fistula (Fig. 3b). However, overall recurrence rate of perianal abscess or fistula-in-ano did not differentiate between the three groups (Table 1).

### Bacteriology from perianal abscess swabs

While Escherichia coli strains were detected frequently in patients from both the DR(−) and DR(+) group in equal proportions, significantly more patients from the DR(+) group had evidences for Streptococci (p = 0.0003), Staphylococci (p = 0.0041), Proteus (p = 0.0043) and Bacteroides strains (p < 0.0001) compared with DR(−) patients (Table 2).

**Figure 4:**
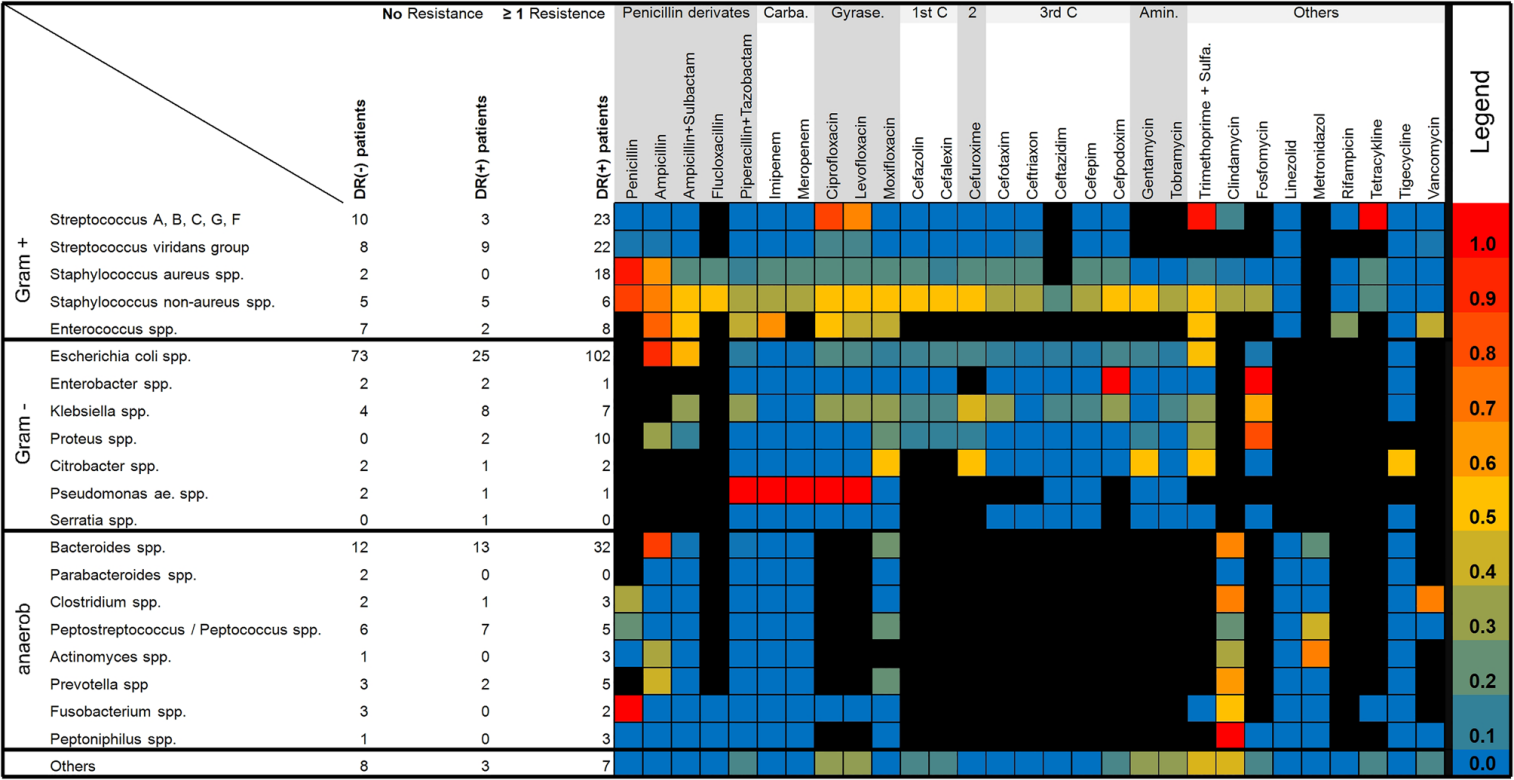
Heatmap of antibiotic resistances in detected isolates. Colored boxes indicate the frequency of detected acquired resistances in the respective bacteria from 0% in blue to 100% in red. Black boxes depict intrinsic resistances in the detected bacteria as indicated by the respective EUCAST (The European Committee on Antimicrobial Susceptibility Testing, http://www.eucast.org) documents. *Carba*. Carbapenem, *Gyrase.* Gyrase inhibitor, *1st C., 2, 3rd C.* 1st, 2nd, 3rd generation cephalosporin, *Amin*. Aminoglycoside, *Sulfa*. Sulfamethoxazole.

The heatmap in Fig. 4 gives a systematic overview regarding the detected microorganisms and observed acquired drug resistances in our patient cohort. Beneath the formally known and very common resistances against penicillin derivates in *Staphylococci*, *Escherichia coli* and *Baceroides* sp. as well as resistances against fluoroquinolones in Streptococci and Staphylococci, there were some other acquired resistances observed in microorganisms from perianal abscess cultures against other widely used antibiotics in general surgery like cotrimoxazole (i.e. Trimethoprim + Sulfamethoxazole) and Clindamycin in the present patient cohort. Furthermore, there are some frequent and worrisome acquired drug resistances against first and second generation cephalosporins, especially Cefazolin and Cefuroxime as well as Metronidazole in the context of perianal abscess and fistula surgery, as these drugs are routinely administered for the purpose of perioperative single-shot antibiotic prophylaxis (Table 2, Fig. 4).

### Correlation analysis

**Figure 5:**
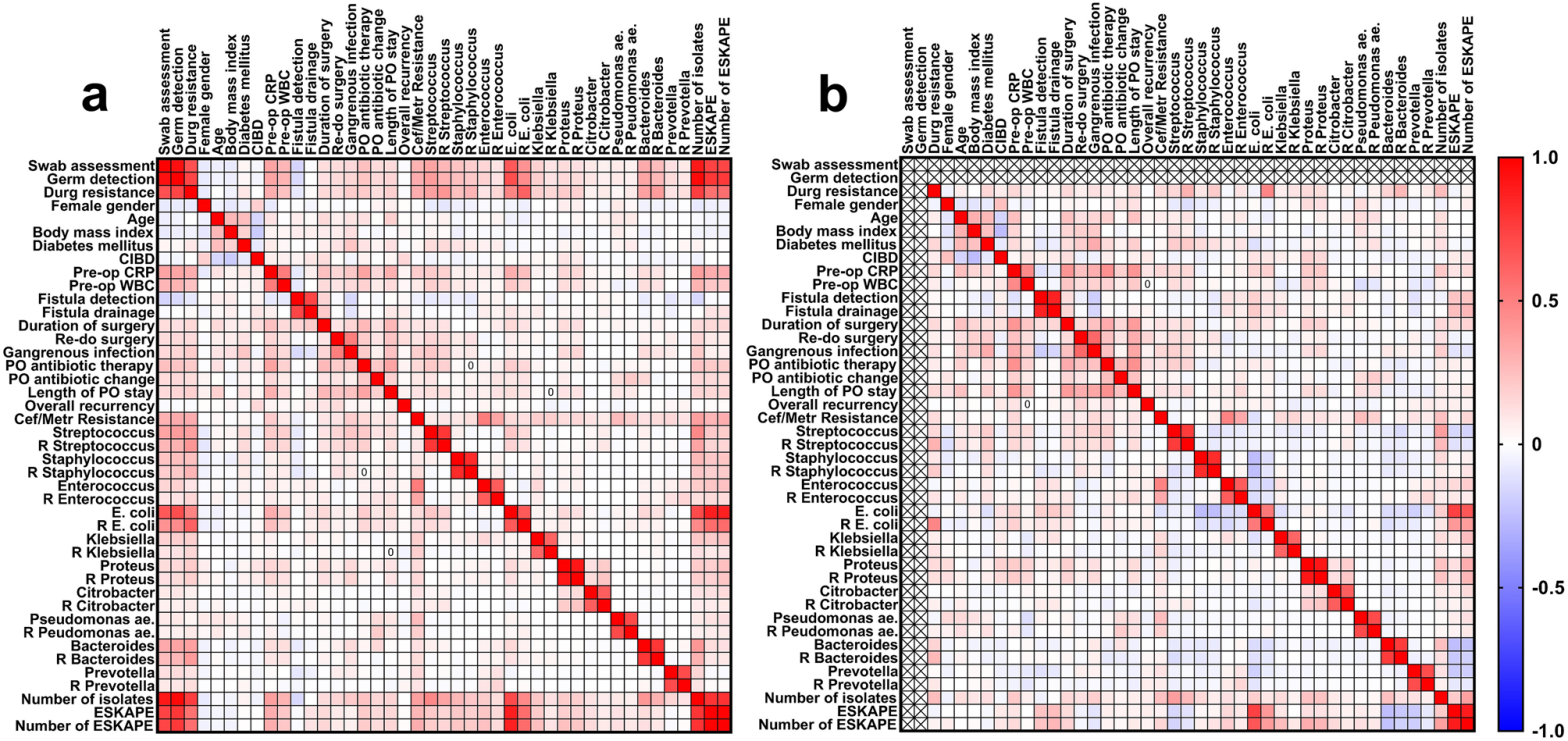
Correlation heatmaps between perioperative outcomes and bacteriology. Correlation analysis was performed either in the whole patient cohort (**a**) or only in patients with intraoperative swab assessment and consecutively microbiological examination of purulence from perianal abscesses (**b**). Color in the boxes indicate the respective Spearman’s rank correlation coefficient (r_SP_) from − 1.0 in blue to + 1.0 in red. *CIBD* Chronic inflammatory bowel disease, *CRP* C-reactive protein, *WBC* White blood cell count, *PO* Postoperative, *Cef/Metr* Cefuroxime/Metronidazole, *R* Resistant, *ESKAPE* *Enterococcus faecium*, *Staphylococcus aureus*, *Klebsiella pneumoniae*, *Acinetobacter baumanii*, *Pseudomonas aeruginosa*, *Enterobacter* sp. (although not intended originally to classify community-acquired infections, the ESKAPE definition was used for this analysis).

Correlation analyses confirmed results from the group comparisons. In the whole cohort with 817 patients, drug resistances of the detected bacteria correlated significantly with preoperative WBC counts (r_SP_ = 0.24, p < 0.0001), CRP values (r_SP_ = 0.31, p < 0.0001), with the presence polymicrobial culture (r_SP_ = 0.75, p < 0.0001) and ESKAPE pathogens (r_SP_ = 0.55, p < 0.0001) as well as with the need for postoperative antibiotic therapy (r_SP_ = 0.20, p = 0.0001), length of postoperative in-hospital stay (r_SP_ = 0.18, p < 0.0001) and re-do surgery during short-term follow-up (r_SP_ = 0.14, p < 0.0001), but not with fistula drainage or overall recurrence rates (Fig. 5a).

In patients with microbiological examination of intraoperatively harvested perianal abscess swabs a weak correlation was found between diabetes mellitus and the detection of Staphylococci (r_SP_ = 0.14) and Streptococci (r_SP_ = 0.19) from abscess swabs as well as between chronic inflammatory bowel disease and proteus species (r_SP_ = 0.18, all p ≤ 0.01). The detection of streptococci correlated significantly with the need for re-do surgery during short-term follow-up (r_SP_ = 0.14, p = 0.0092). However, detection of ESKAPE species and especially enteric bacteria, including Escherichia coli and Enterococci, correlated with intraoperative detection of fistula (r_SP_ = 0.22, p < 0.0001; r_SP_ = 0.18, p = 0.0013 and r_SP_ = 0.11, p = 0.05, respectively), requiring fistula drainage procedure (r_SP_ = 0.26, p < 0.0001; r_SP_ = 0.21, p = 0.0001 and r_SP_ = 0.11, p = 0.04, respectively, Fig. 5b).

## Discussion

Perianal abscess is an extraordinary frequent disease in general surgery. Although the majority of patients reported here suffered—as retrospectively estimated—from rather simple and uncomplicated disease, presented data suggest with approximately 27% of all patients an alarming high rate of acquired drug resistances in bacteria detected in purulence swabs from perianal abscesses obtained during surgical drainage procedures especially in patients with a complex and more severe disease. A precise register of bacterial strains together with analyses of drug resistances is presented in the heatmap of Fig. 4. To the best of our knowledge this is the first publication providing a detailed microbiological analysis including the most frequent intrinsic and acquired drug resistances in this common disease. By encompassing even intrinsic resistances and considering local differences in the resistance profile of the reported microorganisms, the resistance heatmap provides guidance for an adequate and effective antibiotic therapy, if necessary, in the acute care setting with abscess drainage as well as in situations of re-do surgery or an intended staged surgical approach for fistulectomy. While some authors advocate empiric antibiotic usage following surgical drainage of perianal abscesses, which may avoid fistula-in-ano formation^17–19^, this was not the clinical standard in the present patient cohort. However, there were some patients reported here, especially those with a more severe disease, who required antibiotic therapy even after surgical abscess drainage. Of note, 39.5% of the patients with an observed drug resistance in bacteria from perianal abscess swabs continued postoperatively with antibiotic therapy and ten patients of them needed changes in the antibiotic regimen following bacterial culture results. Both the high incidence of drug resistances observed in microbiological examination as well as high rate of antibiotic therapy after surgical abscess drainage reflect the effectiveness, clinical relevance and therapeutic impact of microbiological testing of purulence swabs from perianal abscesses especially in patients with a complex and more severe disease or other risk factors for poor outcome. The latter might include diabetes mellitus, chronic inflammatory bowel disease and immuno-suppression, as microbiological examination from perianal abscesses of patients suffering from these diseases are frequently accompanied by different, but characteristic cultural results apart from the “classical” cryptogenic perianal abscess^7,8,11–13^. Furthermore, a longer duration from index surgery with abscess drainage until definitive fistula repair was shown in the present study especially for DR(+) patients with detected fistula-in-ano. This may be a result of prolonged perianal inflammatory state and consecutively impaired wound healing in these patients. Thereby, patients undergo single-shot antibiotic prophylaxis upon any kind of fistula repair, vice versa we have shown in the present analysis of antibiotic susceptibilities an alarming high rate of drug resistances against the commonly used antibiotics for peri-surgical single-shot prophylaxis, including cefazolin, cefuroxime and metronidazole. If an appropriate short-term antibiotic therapy following surgical perianal abscess drainage, as recommended recently by some authors^17,18^, can not only reduce the development of fistula-in ano but also can reduce the duration until definitive fistula repair, should be clarified by further prospectively conducted trials. Short-term antibiotic therapies following abscess drainage surgery as well as perioperative single-shot antibiotics for a second surgical procedure even in long-term follow-up should therefore be guided by the microbiological examination of pus obtained from the initial abscess to adequately enhance their effectiveness. Nevertheless, overall recurrence rates of perianal abscess seem to be independent from microbiological results obtained from pus swabs during the index surgery^1,8^.

Beneath different strains of Cocci, coliform bacteria and Bacteroides sp. were most commonly detected in perianal abscesses of patients from the present study. Interestingly we found a significant amount of community-acquired drug resistant bacterial strains especially for coliform bacteria, Bacteroides and Cocci, which would not be expected upon review of the current literature^12^ and we reported some significant correlations between the detected microorganisms and perioperative patient care. Therefore, detection of enteric bacteria correlated with the finding of a fistula-in-ano during index surgery, which may indicate a cryptoglandular origin of the infection^14,20,21^. However, whether the bacteriology allows conclusions regarding the etiology (non-cryptoglandular versus cryptoglandular origin with fistula-in-ano) of perianal abscesses remains elusively from the current literature^7,8^. Staphylococci as well as Streptococci isolates were associated with diabetic patients in the present study. Diabetes mellitus was highlighted as a special disease entity for patients with perianal abscesses by Alabbad et al. and Liu et al. and, in contrast to our correlation analysis, the latter described a special bacteriology, including Klebsiella pneumoniae as the predominant pathogen in purulence from abscesses of these patients^12,22^.

Although the evidence is low, the routine assessment of intraoperative swabs from perianal abscesses and investigation of its bacteriology is controversially discussed in the current literature and had even been advocated as being unnecessary, not useful and not cost-effective by some authors^8,9^. In this line, Leung et al. and Shaughnessy et al. questioned the influence on clinical effectiveness of treatment modalities in patients with perianal abscess through results of intraoperatively obtained pus swabs because they had not found any impact of microbiological results on perioperative patient care^7,23^. Even Lalou et al. do not recommend routine microbiological examination of pus swabs from uncomplicated perianal abscesses obtained during surgical drainage procedure, however, they concluded to change that paradigm in patients with a more complex disease including immuno-compromised status, extensive soft tissue infection or severe gangrene, and signs for perianal or systemic sepsis^10,13^. Also Albright et al. reported a high detection rate of methicillin-resistant Staphylococcus aureus in patients with a more severe disease including extensive perianal induration or erythema^14^, underlining the relevance of microbiological testing of abscess swabs in these patients. This may be highly dependent on the setting, though, as the prevalence of methicillin-resistant Staphylococcus aureus varies widely between patient populations. In this line, differences in characteristics and outcome of the three groups reported in the present study allow a dedicated view on the clinical impact of microbiological examination of perianal abscesses. Assessment of swabs from pus of perianal abscesses is not necessarily the clinical standard during drainage surgery and is basically left to the experience and opinion of each single surgeon in our department. From the clinical experience, swabs are routinely taken from patients with more severe disease, but not necessarily from uncomplicated abscesses. This is logical, because in cases of uncomplicated, simple and well loculated perianal abscesses excluding surrounding cellulitis and tissue infection the results of microbiological testing lack relevance in postoperative patient care after adequate surgical abscess drainage^1,7–10^. As our data suggest, the disease of patients from the No_Swab group was, as retrospectively anticipated, milder and not as severe as of patients from both other groups. Preoperative length of hospital stay (i.e. duration from presentation to surgery), pararectal and supralevatoric abscess localization^3,24^, presence of gangrenous soft tissue infection, necessity of protective stool deviation, duration of index surgery, preoperative CRP values as well as WBC counts were used as parameters for retrospectively estimating disease severity between the groups. Differences in disease severity are also reflected by perioperative outcome of patients from the three groups, including postoperative length of in-hospital stay and even duration from index surgery until fistulectomy. Our data prove the clinical relevance and impact on perioperative patient care of microbiologic testing especially in patients with a more severe perianal disease. These patients in particular carry the high risk of multiple drug resistances in their multi-bacterial cultures from perianal abscess purulence. However, a major point of criticism is the delayed availability of microbiological culture results with resistance testing resulting in ineffectivity regarding the impact on postoperative patient care through discharge and failure to follow-up of patients with an uncomplicated disease on the one hand^7–10^ as well as a consecutive delay to an adequate antibiotic therapy after surgical drainage in patients with a complicated and severe disease probably with systemic sepsis on the other hand. If novel methods for rapid, bed-side microbiological point-of-care diagnostics, e.g. NAAT (Nucleid Acid Amplification Test)-based or molecular detection of bacterial DNA as well as resistance genes or real-time metagenomics sequencing approaches^25–27^, absent from classical cultural isolation of pathogens, can be suitable and cost effective for patients with more complicated perianal infectious diseases and can improve the time to an adequate antibiotic intervention after surgical drainage especially in patients with extended perianal soft tissue infection or gangrene as well as signs of systemic sepsis and patients who are in favour to develop poor outcome upon perianal infection might be the basis for further prospectively conducted trials^1,5,28,29^.

Nevertheless, beneath the retrospective character, the retrospective estimation and assessment of disease severity between the three groups as well as the long observation period are the strongest limitations of the study. In this line, the individual intraoperative collection of swabs with consecutive microbiological examination may be considered as a limitation of the retrospective analysis. As extensively stated before, intraoperative swabbing was undoubtedly a clinical decision at the discretion of the surgeon and was not performed in all cases. Thus, clear conclusions on the exact germ spectra and exact incidences of drug resistant pathogens are not possible based on the data presented here. However, our data and the antibiotic resistance heatmap give an unique overview on bacteriology in complicated perianal abscess cases as well as important implications for perioperative treatment in daily clinical routine. Another limitation may be the duration until definitive fistula repair as the surrogate outcome in longer-term follow-up. As this was the longest in DR(+) patients, this parameter is only an indicator for severity of the initial infection and the duration how long the infection took to settle down. It should not be regarded as an ultimate outcome for overall treatment success in the long-term follow-up. For the latter, we did not observe differences in the overall recurrence rate between the patient cohorts. However, the current study gains the evidence for microbiological testing and bacterial findings from perianal abscesses as well as the respective drug resistances in those bacteria and forms hypotheses for further prospectively conducted research in that field.

## Conclusion

In conclusion, drug resistant bacteria in perianal abscesses are frequent and relevant in patients with complex diseases, in cases of severe or even gangrenous infection of surrounding perianal tissue, higher located fistula, supralevatoric and pararectal abscesses. Knowledge of the germ spectrum as well as drug resistances is useful to guide subsequent perioperative therapy in these cases. This should be considered by the treating surgeon with utmost care. Based on results of the present study, patients who may benefit sufficiently from rapid microbiologic testing of pus from perianal abscesses are those with severe perianal and perineal phlegmonous or gangrenous infection alongside with the abscess and patients with fistula-in-ano who very likely have to undergo re-do surgery for re-debridement or fistulectomy in short- or long-term follow-up. The beneficial effects of intraoperative pus swabs during index abscess drainage surgery in these patients have to be evaluated in further prospective trails.

## Data Availability

The datasets used and/or analyzed during the current study are available from the corresponding author on reasonable request.

## Abbreviations

EUCAST: The European Committee on Antimicrobial Susceptibility Testing
CRP: C-reactive protein
WBC: White blood cell
DR: Drug resistant
r_SP_: Spearman’s rank correlation coefficient
ESKAPE pathogens: *Enterococcus faecium*, *Staphylococcus aureus*, *Klebsiella pneumoniae*, *Acinetobacter baumanii*, *Pseudomonas aeruginosa*, *Enterobacter* Species
NAAT: Nucleid acid amplification test
